# A longitudinal qualitative evaluation of the rollout of a complex digital intervention across healthcare organisations and the significance of implementation pace

**DOI:** 10.1186/s12913-026-15183-w

**Published:** 2026-07-21

**Authors:** Louise Laverty, Paul Wilson, Darren Ashcroft, Niels Peek

**Affiliations:** 1 School of Dentistry, University of Leeds, Leeds, UK; 2 School of Primary Care and Health Services Research, University of Manchester, Manchester, UK; 3 School of Pharmacy and Pharmaceutical Sciences, University of Manchester, Manchester, UK; 4 The Healthcare Improvement Studies Institute, University of Cambridge, Cambridge, UK

**Keywords:** Implementation, Infrastructure, Technology, Pace, Health organisation, Evaluation

## Abstract

**Background:**

The Safe Medication Dashboard (known as SMASH), an intervention to reduce hazardous prescribing in general practice through a pharmacist-led intervention and web-based dashboard, was piloted in 2017. A rollout of SMASH by the regional health innovation network was funded in 2019.

**Methods:**

A longitudinal qualitative evaluation of the regional implementation effort was conducted over three years, including in-depth semi-structured interviews with relevant stakeholders (*n* = 18), non-participant observations (*n* = 42), and documentary analysis.

**Results:**

Assumptions about the replicability and complexity of the intervention led to an implementation strategy and pace that were misaligned. The implementation of SMASH suffered from delays, diversions, and disruptions due to unanticipated infrastructure needs, regulatory changes in data sharing, and the health systems’ response to the COVID-19 pandemic.

**Conclusion:**

The findings will be discussed alongside relevant implementation frameworks to highlight how interdependencies between wider policies, organisations, and technologies can significantly shape the trajectories and pace of implementation efforts.

**Supplementary Information:**

The online version contains supplementary material available at https://doi.org/10.1186/s12913-026-15183-w.

## Background

In the context of rising demands and costs in healthcare provision globally, the implementation of innovations is viewed as a route to maximise efficiency and improve care at a rapid pace [1, 2]. Implementing innovations that have been successful elsewhere provides policymakers with a risk-averse approach to bringing about change in these ‘fast’ policy climates [3, 4]. However, extensive research on the diffusion of interventions shows that the translation of innovations into practice is rarely straightforward [5, 6]. Research in the social sciences and organisational studies reveals the complex and situated nature of implementation efforts, involving relational, material, organisational, and political factors [for example, 7, 8, 9]. Governments in several countries, recognising the complexity of translating knowledge into healthcare, have been funding and developing innovation organisations to support healthcare innovation over the past decade [10].

In the UK, innovation organisations known as Health Innovation Networks (HIN, formerly Academic Health Science Networks) now form part of the health care landscape. They receive government, NHS, and external funding to develop more streamlined pathways to adopting innovations by reducing translation barriers [10, 11]. These established innovation organisations work as a non-hierarchical network, which, in addition to delivering national policy priorities, can operate across organisational and temporal boundaries to generate evidence across the translation pathway [10, 12]. This paper reports on a longitudinal qualitative evaluation of the rollout of a digital intervention by one of these Health Innovation Networks in England, UK. The evaluation focused on the meso-level (structural and organisational) enactment of the rollout, looking at the judgments and actions of decision makers under particular circumstances and contexts [6]. In what follows, key concepts related to innovation implementation and digital implementation frameworks will be introduced to frame the paper, alongside a description of the digital health intervention itself.

### Implementation of innovations

Digital technologies are regarded as fast, efficient and adaptive, and as such, their introduction is assumed to be a solution to rising healthcare demands. Policy discourses emphasise the importance of implementing innovation at scale and pace [3, 6] but often fail to define these terms. Looking at the former, scale is closely linked to spread and sustainability. Greenhalgh and Papoutsi [13] define spread as the replication of an innovation in a different setting. Scale is defined as the development of infrastructure to support innovation, and sustainability is the embedding of innovation into everyday practice [13]. Papoutsi, Greenhalgh and Marjanovic [14] argue that spread, scale, and sustainability should be considered as interdependent and overlapping rather than linear.

Compared to spread, scale, and sustainability, however, pace as a concept is underexamined in implementation literature [6]. Pace has been defined as the speed, density, rhythm and tempo of activities [15]. In policy, pace is assumed to be singular and linear, with *fast* policy and *fast* implementation considered preferable to the slow inertia often associated with healthcare change [16]. Touhy [6] warns that both fast and slow paces can create problems. A fast pace can create pressure to act and cooperate but may lead to conflict. A slow pace can allow time to develop trust and manage emerging problems but may suffer if priorities change.

The approach to pace, here, draws on a social science approach to time that recognises that in practice, pace is constantly in flux, being compressed, extended, and evolving in response to local contexts, political pressures and crisis management [3, 4]. Literature in this field suggests that people are active participants in pace, shaping and adapting time to different tasks and needs, although choices are constrained by circumstances [4, 17]. This approach to understanding the significance of pace can illuminate both how the role of active decisions around strategies and allocations of resources and wider social constraints may impact on implementation.

### Implementation framework

Several frameworks and theories have been developed to make sense of implementation efforts in healthcare [9, 18], including several focused on digital interventions [8, 19]. This reflects the fact that despite the promise of much-needed time efficiencies and the opportunity to accelerate change through digital innovation, they are rarely adopted at scale or pace within health organisations [4, 20]. This paper draws on the NASSS framework, which examines the **n**on-adoption, **a**bandonment, and challenges to **s**caling up, **s**preading, and **s**ustaining technology-supported change in health and social care [8]. The NASSS framework is theory-informed and developed based on the synthesis of rich qualitative work on digital innovation in healthcare settings. It aims to explain the complexity and unpredictability of implementing digital innovations in healthcare across various actors, infrastructure, organisations, and systems. There are seven domains in the NASSS framework, asking for consideration of the (1) condition, (2) technology, (3) value proposition, (4) adopter system, (5) organisation, (6) wider context, and (7) how digital technologies are embedded over time [8]. The NASSS was chosen over other implementation frameworks due to the focus on technological innovations and the domains felt to be better suited to examining meso- and macro-level factors. It was utilised in the planning of the evaluation and topic guide, with the relevant domains revisited in the discussion to contextualise the findings.

### Overview of the intervention

NHS England commissioned the 15 regional Health Innovation Networks (HIN) to roll out a pharmacist-led information technology intervention (PINCER) across England in 2019. The PINCER intervention has pharmacists working collaboratively with primary care clinicians to act upon medication data (social component), from a set of computerised queries to identify ‘at-risk’ patients (technical component), to reduce potentially hazardous prescribing [21]. The Health Innovation network in Greater Manchester, however, chose to roll out a locally developed adaptation of PINCER, called the Safe Medication Safety Dashboard (SMASH). SMASH adopts the same indicators and social component as the PINCER intervention, but with a different technical component: a web-based interactive safety dashboard linked to electronic patient records in primary care, updated daily. SMASH was piloted in the city of Salford between 2015 and 2017 and showed a reduction in potentially hazardous prescribing [22] with pharmacists able to incorporate the dashboard into their routine work in primary care [23]. This offered the Health Innovation network the opportunity to fulfil the national commission whilst demonstrating their ability to introduce a locally developed, more digitally innovative intervention.

This paper examines the regional implementation of SMASH as a complex digital intervention, focusing on the role of the Health Innovation network and the pace of implementation. The evaluation offers a shift in perspective away from the micro-level, where end-users work to implement an intervention, to the meso- and macro-level work done by decision-makers. This approach lends itself to looking at what Peck and Theodore [3] has called the ‘sticky reality of delivery’. Over three years, the longitudinal qualitative study included interviews, non-participant observation, and documentary evidence to address the following research questions:

1. How was the intervention spread across the different localities in the region?
2. What regional and organisational infrastructure and support were needed to implement and sustain the intervention across different localities?
3. What were the factors that, in the experience of local stakeholders, contributed to or detracted from the uptake of the intervention?

## Methods

### Methodology

The study used an interpretive, sociotechnical, approach that recognises that the implementation of technology has social and organisational dimensions beyond the technology. The evaluation was planned concurrently, and the qualitative data collection was focused on the meso-level organisational enactment of macro-level policy and planning, whilst considering the interactions with micro-level local organisations. The longitudinal approach allows an exploration of how decisions are negotiated and given meaning in social practices over time [24, 25].

### Design and setting

Data collection started in April 2020, with the collection of documentary evidence and non-participant observation of the delivery and implementation meetings hosted by the regional Health Innovation network (hereafter HIN). Subsequently, the researcher conducted non-participant observation of other relevant meetings, such as regional information governance (IG) meetings, online training with localities, and strategic clinical meetings related to the implementation of SMASH until May 2023. Relevant stakeholders, identified through observations and team discussion, were invited to take part in interviews at two time periods:

1. Spring 2021, at the end of the funded one-year implementation activity.
2. Winter 2022, as the intervention was being integrated into another system.

Fieldwork and interviews were initially planned to be done in person, but moved online due to the COVID-19 pandemic.

### Procedure

Ethical approval was granted by the NHS Health Research Authority (reference 20/HRA/1062). Community consent was considered appropriate for the non-participant observations, given the large and changeable number of staff attending various meetings and that meetings were minuted for public record. Before the first observation of a meeting, an information sheet was sent to attendees to introduce the researcher and provide information on how to opt-out. This was reiterated at the start of meetings, and attendees could message the researcher directly. The researcher observed 42 relevant meetings and took anonymised fieldnotes. 166 pieces of documentation (such as meeting notes, agendas, business cases, and communication briefings) were collected that had been shared by the implementation leads or were publicly available on organisational websites over the three years.

Sampling was purposive, attempting to get the perspective of local and regional stakeholders in different roles relevant to implementation across the time points. Relevant stakeholders were identified through fieldwork and invited to participate by email. If they agreed to participate, they were sent an information sheet and consent form before a time and date for an online interview was arranged. Key stakeholders were reinterviewed, where possible, at the second interview phase. 18 semi-structured interviews took place with individuals from the HIN, local governmental and NHS organisations, and several small and medium enterprises (SME), as can be seen in Table 1. Sampling was guided by the concept of informational power [26] to focus on those directly involved in any aspect of the implementation of the work, and methodological triangulation with the observations. A topic guide was developed by drawing on relevant literature, including the NASSS framework, and was adapted after initial interviews and fieldwork to ensure it captured the appropriate information (please see supplementary materials). The interviews were conducted by an experienced female qualitative researcher who was external to the implementation team but familiar to most participants from observations of meetings. Interviews lasted between 37 and 87 min (average 55 min). Interviews were digitally audio-recorded with permission using an encrypted device and transcribed by a university-approved supplier. Due to the risks of participant identification due to the specifics of the innovation, the quotes refer solely to job roles rather than organisations.

**Table 1:** Participant job roles and interview time

Interview	Time	Job Role	Organisation	Organisation level	Repeat
1	1 (Spring 2021)	Programme Director	Internal	Regional	
2		Programme Lead	Internal	Regional	
3		Project Manager	Internal	Regional	
4		Clinical Lead	Internal	Regional	
5		Project Manager	External	SME	
6		Information Governance Lead	External	Local	
7		Information Governance Lead	External	Local	
8		Medicine Optimisation Lead	External	Local	
9		Medicine Optimisation Lead	External	Local	
10		General Practitioner	External	Local	
11		General Practitioner	External	Local	
12	2 (Winter 2022)	Clinical Lead	Internal	Regional	Yes
13		Project Manager	Internal	Regional	Yes
14		Medicine Optimisation Lead	External	Local	
15		General Practitioner	External	Local	
16		Information Governance Lead	Internal	Regional	
17		Software Engineer	External	SME	
18		IT Lead	External	Local	

### Data analysis

The data collected was stored and managed in NVivo software. Data analysis was inductive and followed thematic analysis processes of familiarity, line-by-line coding, initial grouping of codes (by the qualitative researcher), reorganisation of themes, and development of interpretative themes within the wider research team [27].

## Results

### Theme 1: Implications of decision-making driven by assumptions

This theme reflects on how assumptions about the replicability, adaptability, and complexity of the national PINCER intervention and rollout shaped decision-making about SMASH and its subsequent implementation pace.

#### The importance of simplicity and adaptability to the pace of the national rollout

Understanding the background of the national rollout of the PINCER intervention can provide context for decisions regarding the regional implementation strategy and the pace of SMASH. The timeline of the development of the PINCER intervention to national rollout, as is common with the pace of translation of research evidence into clinical practice, was sixteen years. Published evidence (pilot trial in 2003, full trial in 2012), alongside the inclusion in national clinical guidance in 2018, resulted in national funding in 2019. Relevantly, within this time, funding was awarded to develop PINCER as a social franchise model. This approach leans on the replicability of an evidence-based intervention to develop a standardised package of training and delivery to better scale it across the healthcare system. The simple technological component of the intervention, according to the NASSS framework, meant PINCER was particularly suited to this approach.

The simple, replicable nature of PINCER also meant it was relatively adaptable. Firstly, in adapting to the change of pace due to national funding. An incremental scale-up of PINCER was already underway in one region when the national policy push and the injection of funding meant that the implementation plan had to change to a ‘big bang’ approach, accelerating the pace to meet the compressed timelines as a result of time-limited funding:

The idea would have been that we would have had a couple of early adopter sites. And then we might have had two or three regions coming on board each year. So that eventually, sort of after a period of time, all the regional health innovation networks would have come on board. But of course, then NHS England kind of said that they wanted all the regional health innovation networks to sign up for PINCER, so it became one of their priority areas, and they wanted it all done in the first year. So then, suddenly, we went from sort of having one or two early adopter sites to having all 15 regional health innovation networks signed up. *Interview*,* Clinical Lead*,* Time 1 (Spring 2021)*

Secondly, the social franchising model approach of the PINCER intervention meant that they could adapt relatively easily to the challenges of remote working that came about as a result of the COVID-19 pandemic:

What we did was we turned that into an E-learning tool. So, that went online as a kind of self-directed learning tool. And then we actually changed the other two sorts of action learning sets into a series of self-directed learning, but also facilitated webinars. *Interview*,* External Project Manager*,* Time 1 (Spring 2021)*

#### Meso-level decisions to diverge from the national plan

There were two rationales for the regional HIN deciding to diverge from the national rollout by adopting SMASH instead of PINCER. Firstly, it offered the regional HIN an opportunity to demonstrate its commitment to fast, accelerated digital innovation developed within the local health system. SMASH was developed with local academic partners to develop a more interactive technological interface of the PINCER intervention and was piloted within the region [22, 28]. As one of the regional HIN’s first national commissions, adopting SMASH as their version of the PINCER intervention, was seen to help evidence its ability to behave differently from the other HINs:

We have the opportunity here, I think, to do something more innovative, to achieve the same thing. But not just achieve the same thing, but to enhance it. *Interview*,* Internal Programme Director*,* Time 1 (Spring 2021)*

Secondly, SMASH was seen to be an easier sell for the HIN to local partners. The evaluation of the pilot suggested that the interactive nature of the SMASH dashboard meant more regular engagement and could lead to greater sustainability of the intervention [23]. The sustainability of SMASH alongside the novel technological component of the intervention was perceived to be of greater value to local partners:

When we were presenting the PINCER project to local Medicines Optimisation leads, they were saying, well, we’ve done it before, or there are a couple of other tools. You’re just asking us to do the same thing again. So that didn’t go down very well. Whereas, when we brought the opportunity for SMASH into the equation, the conversation turned into a much more positive conversation about seeing the opportunity for ongoing sustainable management of safety indicators. *Interview, Internal Programme Lead, Time 1 (Spring 2021)*

#### Intervention and implementation assumptions at the meso-level

The decision to adopt SMASH, however, was based on several assumptions that would later impact the implementation strategy and pace. The most important assumption was that SMASH was a simple adaptation of the PINCER intervention, as only the technological component was changed. As such, SMASH was understood to be a replicable product that could be spread across localities like PINCER. However, the change of the technological component introduced complexity and interdependencies. The simple audit tool of PINCER is put onto existing local computers in general practice and does not involve any movement of patient data. SMASH, in contrast, is a complex technology that relies on the movement of patient data and thus depends on digital infrastructure, data governance, and data storage on external servers. This was not a problem in the pilot, which was conducted in a digitally mature Trust, but as unfolded over the three years of implementation efforts, this complexity meant that SMASH could not be easily replicated and spread as anticipated:

The impression I got was that we’re going to roll this out because it’s something that’s been done elsewhere. It’s like the spreader, suppose, it’s a carbon copy of what’s been done elsewhere. I think that’s perhaps the bit that they weren’t kind of expecting. I think a lot of it was around how out of the box something is and how easy it is to replicate. *2nd interview*,* Clinical Lead*,* Time 2 (Winter 2022)*

The assumption that SMASH was replicable and spreadable underpinned the resourcing of the implementation, the scheduling, and the strategy itself. The regional HIN network assigned an internal project manager and initially seconded an experienced clinician to lead the work for the funded year. The clinical lead had experience using SMASH and was embedded in the regional medical optimisation network. Their expected role was based on implementing SMASH as a replicable product. It was to engage local services to sign up, support communication work, and provide training to staff and teams. The implementation strategy from the regional HIN during these initial stages of set-up was to implement SMASH across the localities incrementally. The clinical lead planned to work with some early adopters with digital maturity, expecting other localities to follow: “*It was like a domino strategy; you hit less resistance once a couple of people are on board with it*” (Time 1, Spring 2021). The plan was to have all services trained and with access to SMASH by the end of the funded year.

### Theme 2: Delays, disruptions, and diversions

The theme focuses on the delays, disruptions and diversions of pace and progress that were key narratives in the interviews about why SMASH was not rolled out as expected during that first year.

#### Delays: moving the information governance goal posts

The most significant delay cited in the interviews was the complexity in information governance (IG) related to data sharing agreements needed for the SMASH dashboard. The regional HIN recognised IG as a potential risk to the project in early project meetings in 2018 and 2019, and it was a standing agenda item. However, there was no dedicated IG expertise within the project team, and the work was left to the clinical lead to try and manage alongside the engagement and training tasks (see Table 2 for an overview of the implementation tasks, timelines, and challenges).

**Table 2:**
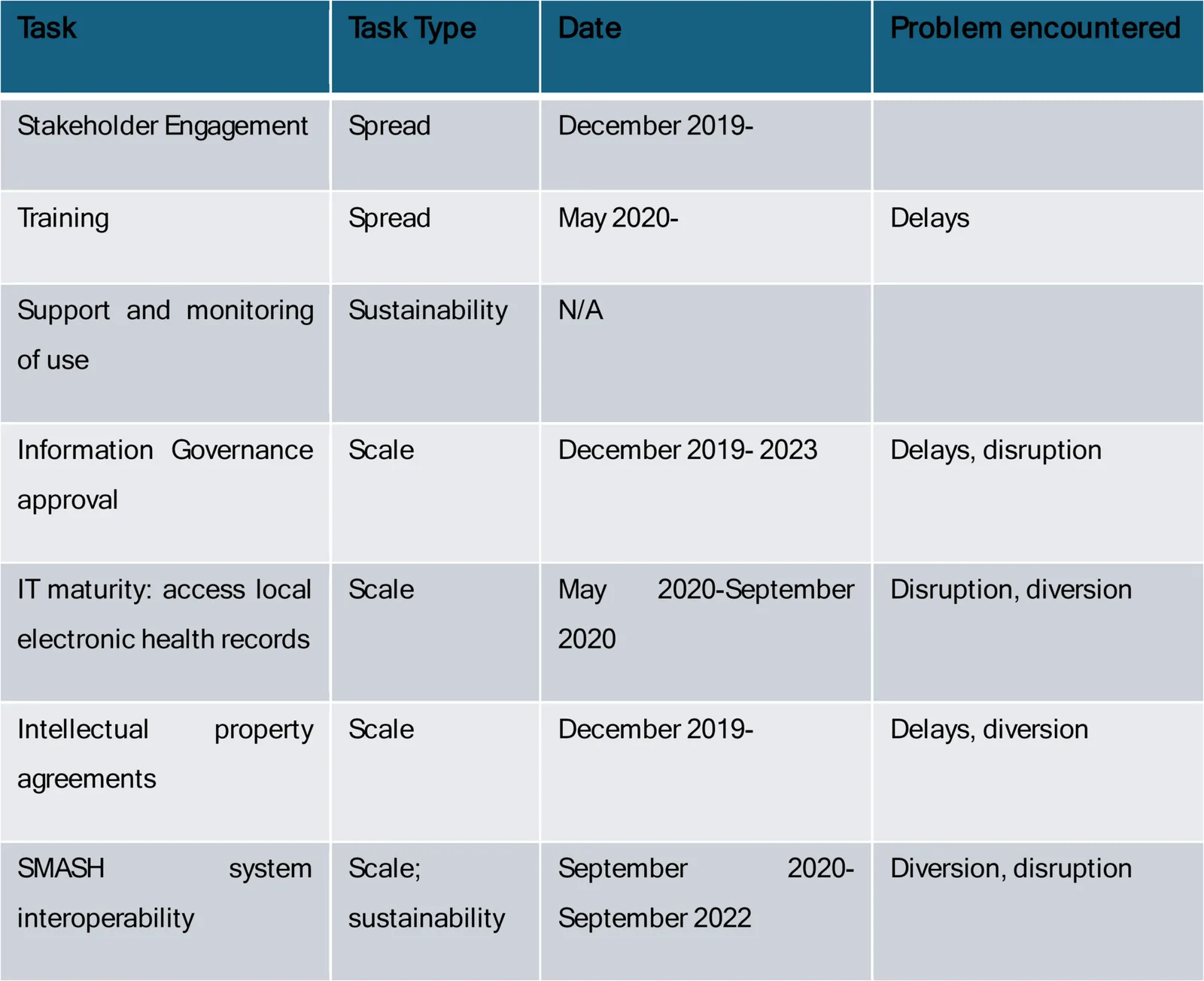
Overview of tasks seen as necessary to implement SMASH

I suppose not being an IG expert but being a clinician with an understanding of IG. And the IT people working with us, again, weren’t experts on clinical governance and data stuff. 2nd *Interview*,* Clinical Lead*,* Time 2 (Winter 2022)*

The complex organisation of health services within the region further complicated this. There were ten localities, ten commissioning groups, ten information governance leads, and hundreds of GPs responsible for patient data that had to approve the data-sharing agreements. Under international and national data protection laws, organisations must conduct a Data Protection Impact Assessment (DPIA) before they start collecting or using people’s personal information in ways that might be risky or invasive. The initial plan was to try to coordinate the development of one DPIA for the whole region that IG leads could sign off on, rather than developing individual DPIAs for each locality:

The DPIA has been created; we need to review the data sharing contract, privacy notices, package docs and briefing documents to be sent to all 10 localities. Hopefully, we can use the existing DPIA. *Health Innovation Network Planning Meeting Notes*,* August 2019.*

The lack of IG standards and infrastructure at the time, however, meant that the regional DPIA was legally not a viable route, and the clinical lead had to do time-intensive work to find and engage with unfamiliar IG leads in each locality to develop individual DPIAs that varied by area with different needs:

The engagement with the IG leads is quite challenging, partially because a lot of time people don’t even know who the IG leads were, what function they had, or whose responsibility it was. And also, people interpret governance completely differently. So, what is fine in one locality is completely not fine in another. *Interview*,* Clinical Lead*,* Time 1(Spring 2021)*

The problem with the NHS as a whole is the fact that, say you’ve got to implement something in an area. That’s eight standards, potentially different standards, to do eight separate DPIAs, to go to eight separate committees, eight separate approvals, and it just becomes an industry when it needn’t be an industry; a lot of things I feel just get far too complicated. We should all have the same standards. *Interview, Information Governance lead, Time 1(Spring 2021)*

#### Wider system disruption: the impact of the COVID-19 pandemic

Six months into the funded year, the clinical lead had engaged with services and started to arrange training while also working on the DPIAs with each locality. However, the start of the COVID-19 pandemic meant widespread disruption. Priorities within clinical teams moved away from quality improvement work like SMASH to managing the pandemic response, and the workforce was redirected, greatly slowing the momentum the clinical lead had been working on. At the same time, the pandemic opened a window of opportunity to change the infrastructure of health and social care data sharing.

Across the NHS, the pandemic was a catalyst for the acceleration of health data sharing. In England and Wales, the government allowed the extended collection and sharing of health and social care data, through temporary legislation, for the COVID-19 response. The development of a regional shared care record was accelerated as part of this response, with all localities brought together through a single external data provider and changing the data sharing infrastructure across the region. This had a significant impact on the SMASH implementation effort. Firstly, all DPIAs written thus far had to be scrapped. Secondly, the DPIAs could not simply be rewritten due to ongoing changes and contract negotiations with the external data provider:

How we might use data sharing and what agreements needed to look like, became very protracted, because this is all going on at the start of COVID. *Interview*,* Information Governance Lead*,* Time 2 (Winter 2022)*

When COVID hit, we were just getting on top of information governance, and we’re just getting ready to be sort of looking at getting it signed off in a few localities, and then the goalposts moved. *2nd interview*,* Internal Project Manager*,* Time 2 (Winter 2022)*

The regional HIN was responsible for commissioning the external data provider for the shared care record, which led to discussions about integrating proven digital interventions, such as SMASH, into the system. The lack of interoperability of SMASH (which was web-based and hosted on an independent server) had always been a reservation for future sustainability. The opportunity to integrate SMASH into the new data provider system, therefore, was welcomed to future-proof the intervention. Rather than continuing, the SMASH implementation was temporarily diverted towards integration within the new data provider system.

#### Diversions: slowing pace for long-term gains

The decision to be integrated into the new data provider system meant compromising progress on rolling out SMASH for future sustainability and led to SMASH becoming dependent on the progress of others:

So, I think the challenge is that SMASH is, you know, a really useful tool. But there’s a whole massive contract negotiation going on. There are always other bigger priorities, and we’re a smaller cog in that machine. And it’s kind of sort of getting in the position of understanding what’s going on with those bigger cogs. So, you can see where our smaller cog fits into the sort of overall picture, basically. *Interview*,* Internal Project Manager*,* Time 1 (Spring 2021)*

This diversion was justified by promising that the delay now would pay dividends later. In particular, the initial incremental implementation plan for SMASH was dependent on the data maturity of different localities, starting with those with integrated digital infrastructure and moving to other localities when they eventually moved to new data systems. This change in infrastructure meant the implementation strategy could shift to getting everyone on board simultaneously:

Due to COVID, the data feeds have changed to the regional shared record. This has delayed the data availability while they change the data infrastructure. This is due imminently, however, and will make the spread much easier as all data will be coming via the same route. *Implementation meeting notes 2021.*

The diversion to inclusion within the external data provider, however, created unexpected complications with intellectual property over the license fees for the PINCER indicators. The licensing fees had been covered for the first year through the national funding. However, the delay and involvement of an external data provider meant decisions around IP and licensing had to move to formal negotiations:

NHS England had funded us for a set period of time to make those indicators available to third parties, for a sort of, you know, a licence at no cost. But obviously, there’s the whole IP issue has to be sorted out and all of that sort of thing. So that’s where the IP and licensing offices comes in. But it just complicated things. Well, when I say it complicates things, it just means that, you know, you have to have those kinds of meetings, and you have to have a licence agreement drawn up, and it has to be agreed, and signed by both parties. So, it just takes time to do those sorts of things. *Interview*,* Internal Project Manager*,* Time 1 (Spring 2021)*

Discussions did include the option of the external data provider buying or licensing the indicators directly as a product. After lengthy discussions, however, the provider dismissed this as not commercially viable, and negotiations had to restart:

I think we got stuck for a long time because of the IP. They own the IP behind a lot of the algorithms as such. So, it got stuck quite a while, that sort of IP kind of discussion. And I think the net result was that it was all too complicated. And it wasn’t worth our effort as a commercial company. To come to any sort of licensing deal, we’ve just kind of said that, perfectly take it on, we’ll happily roll it out, but unfortunately, it wasn’t something we’re going to build to sell elsewhere. Because of the licensing, politics, in particular, was just too challenging. *Interview*,* Software engineer*,* Time 2 (Winter 2022)*

The IP issues became entangled with the build of SMASH into the external data provider systems, with one unable to start until the other was agreed upon. In 2022, an agreement was made, and the build within the external data provider system started. It became clear during this build that new training, sign-ups, and approval processes would be needed for SMASH. A new regional IG lead was appointed during this time, and a new regional DPIA was developed and provisionally signed off for SMASH. By the time these had been completed, however, the licensing agreement had expired again and had to be revisited:

June was expecting to go live but license issues with the new version mean they want to renegotiate it, lawyers and new contracts, delayed *Implementation meeting*,* 2023.*

### Theme 3: Mismatched expectations of pace

This theme focuses on the expectations and experiences of stakeholders on the ground (local leads who were expected to coordinate and manage the intervention in localities) during the SMASH rollout, and how they interpreted and made sense of the delays, disruptions and diversions.

#### The factors shaping expectations of pace on the ground

Stakeholder narratives focused on the disconnect between the pace they were expecting and the reality of being left waiting for the implementation to happen. During the early stages of the rollout, the clinical lead engaged with relevant clinical teams by attending meetings, showing demonstrations of the SMASH dashboard, providing communication materials, and arranging training dates. The stakeholders recalled these meetings and were enthusiastic about SMASH, all articulating the benefits of it:

And SMASH was a useful tool to actually assist the GP with their daily work and draw their attention to some potential problems, so I could understand it straight away. It is a problem that busy GPs have. It was an issue in terms of safety, in terms of optimising the medication patients take. So, it was a great tool was very easy to say yes. *Interview*,* General Practitioner*,* Time 1 (Spring 2021)*

And that’s why SMASH is so good because it’s data-driven, it’s numbers. It’s very objective; it’s not subjective. You can’t say, oh, we’ve done this and nothing happened. You do something, and the numbers move. And nobody can argue with choosing it because it’s patient safety. *Interview*,* Medicines Optimisation lead*,* Time 1 (Spring 2021)*

For the stakeholders, this engagement work set up an expectation that the implementation was imminent. The clinical lead provided remote training to staff in the summer of 2020 at the start of the pandemic. The key question in observed training sessions was when SMASH would be available. The response during a training session in June 2020, from the clinical lead, was that “*registration would be live in the next week and data in a few weeks. Will contact you when it is live*”. As a result, leads in some localities talked about setting up task and finish groups, project groups, and pilots to ensure that the implementation went smoothly with teams.

We thought it would be up and running and ready to launch in May, June 2019. Or June, July 2019. So, in either April or May, we have a half-day intense training and education session. And I got on the agenda to do what’s called the soapbox, so I shared and then walk people through some screenshots of what it would look like what would be involved in it, how the pharmacists will be trained up in using it with the thought that it will be launched a month later, but clearly that didn’t happen. *Interview*,* Medicines Optimisation Lead*,* Time 2 (Winter 2022)*

Stakeholders could guess the various causes of the delays with SMASH; some attributed it to the pandemic, and others knew there had been issues with IG, but mostly, the response was uncertainty:

Why has it not gone further? Certainly, with the DPIA and the data sharing agreement that I reviewed, I was happy with it, and I’d signed it off, I’d given it approval. And I actually assumed it had gone out, but it seems it hasn’t. *Interview*,* Information Governance Lead*,* Time 2 (Winter 2022)*

The initial enthusiasm at the start of the project over the years gave way to cynicism during the 2nd period of interviews that SMASH would ever materialise and that perhaps it would no longer be relevant when and if it did arrive, with trained staff moving on and changes to management and priorities:

I guess just, you know, I tried to get people to be excited about it. Which would be about three years ago now. *Interview*,* Medicine Optimisation Lead*,* Time 2 (Winter 2022)*

I think we’ve got loads of resources he gave us before, because we were already sort of getting ourselves ready for it. I think some of them will need refreshing. Boy, yeah, it’s you know, we missed the boat *Interview*,* General Practitioner*,* Time 2 (Winter 2022)*

**Table 3:**
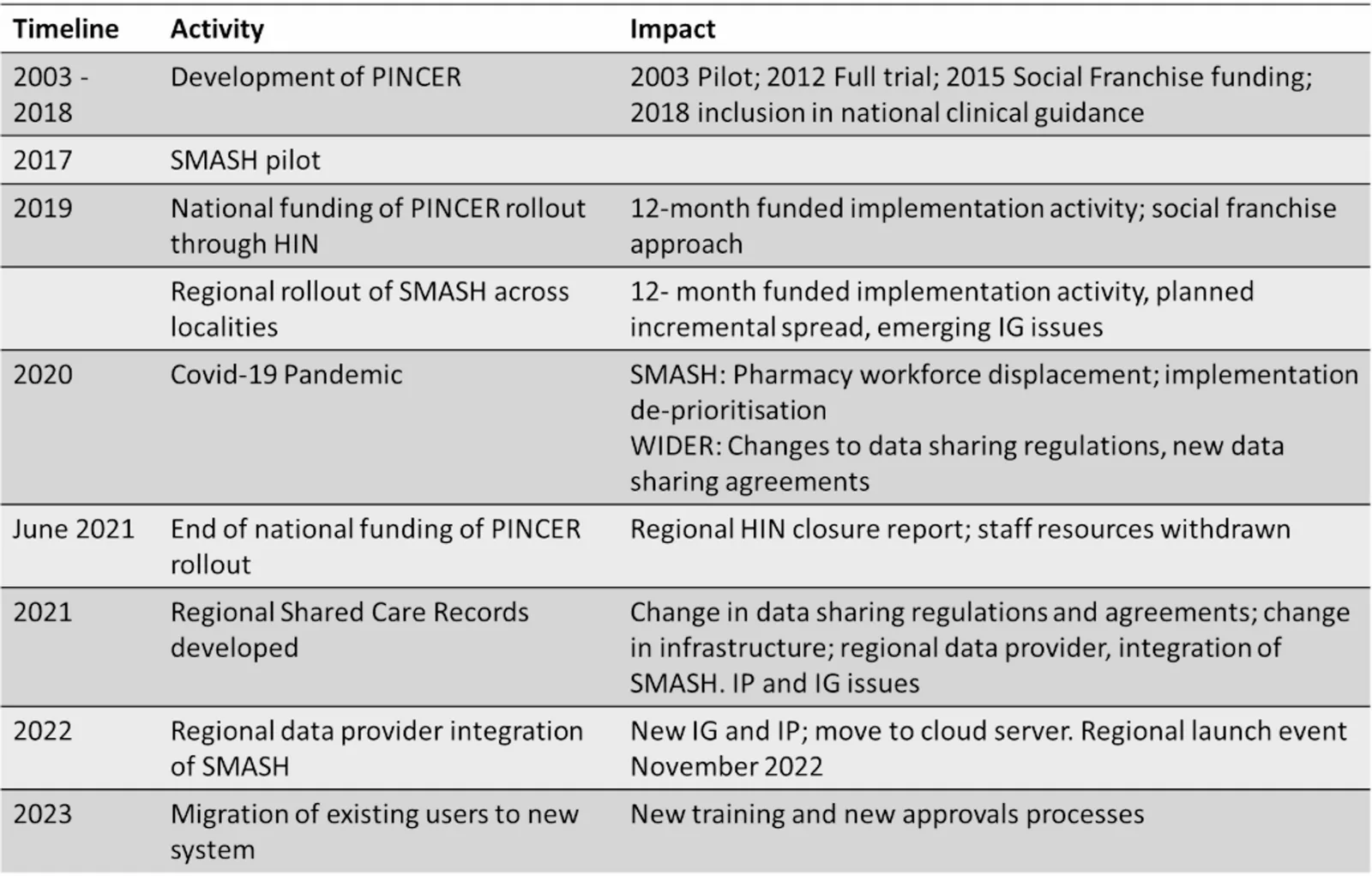
Overview of the PINCER and SMASH implementation timeline

#### Lack of clarity on the role of the regional HIN

Many of the stakeholders discussed the regional HIN’s role when explaining the delays to SMASH. They recognised the value of having a regional organisation for large projects, such as the shared care record, and to negotiate contracts across the region. However, the centralised nature of the work was also seen as a barrier, with no links to the systems into which they were working. Stakeholders reflected on the difficulties those outside the system faced in understanding the fragmented and ever-changing organisation of healthcare in the region.

There was also a mismatch between the timeframes reported by the regional HIN and the lengthy reality of the implementation efforts. In March 2021, at the end of the funded year, the regional HIN produced a closure report and formally stated that SMASH was completed. The report showed three localities as active, having signed off their DPIAs and having access to the old servers, and the rest as pending. This was despite the ongoing negotiations to integrate SMASH into the external data provider. It was also clear that there was uncertainty about whether the localities with access to SMASH were actually using SMASH. For the leadership at this point, sign-up was considered an appropriate measure of implementation. The project manager was reassigned to other work, and the clinical lead’s secondment ended. The regional HIN’s perspective was that their role was to deploy the intervention, which they claimed to have done in the closure report, and then pass it back to the system:

But really, we’re an innovation organisation. Yeah. So, once we’ve designed, developed, and deployed, it’s handed over to the system. We are not the organisation that will maintain an initiative. *Interview*,* Programme Lead*,* Time 1 (Spring 2021)*

Despite this ‘closure’, the project manager and clinical lead continued to work on SMASH alongside other commitments without resources. However, the lack of a formal project team within the regional HIN was felt by stakeholders, who talked about feeling that there was no one responsible for updating them on progress or actioning SMASH:

It’s like nobody knows who’s responsible. Nobody. I mean, that may be just an impression. And all I know is that it’s not me. But that doesn’t mean I know who is. *Interview, Medicines Optimisation Lead, Time 2 (Winter 2022)*

At the end of 2023, SMASH had been built into the new data system, and the new processes and approvals were said to be in place, with the hope of having the new SMASH launched in 2024. Table 3 shows an overview of the timeline of the rollout.

## Discussion

This article presents the findings from a longitudinal, qualitative evaluation of a regional innovation network’s attempt to implement a sociotechnical intervention across a region. Focusing on the meso- and macro-level factors and decision-making that shape the pace of the implementation offers a novel perspective to the existing literature on digital implementation in healthcare. The findings will be situated in relation to the existing literature on pace and the relevant domains of the NASSS framework to more closely explore the key findings. Secondly, the broader implications of the findings for the field will be considered.

The main aim of innovation organisations, such as Health Innovation networks, is to coordinate and aid the translation of innovation interventions into clinical practice, in other words, to improve the pace. The findings here suggest that this cannot be assumed. In this case, pace was particularly impacted by decisions around the implementation strategy, lack of clarity around the role of the organisation, and wider system changes outside of their control, which meant that after three years of activity, the implementation of SMASH was still in progress. However, drawing on social science approaches to time, the findings show that the pace was not simply slow. Instead, pace was non-linear and interdependent on choices made by decision-makers, organisational priorities and wider forces that could accelerate, compress, slow or cause stasis in efforts that had implications for expectations amongst different stakeholders.

Papoutsi, Greenhalgh and Marjanovic [14] suggests that the spread, scale, and sustainability of interventions should be considered as overlapping and simultaneous processes. However, this risks underestimating the role of pace, as the findings here suggest that spread, scale, and sustainability processes can have different temporal dimensions that should be considered. SMASH was considered an adaptation of PINCER, and as such, decision-making around resourcing and implementation strategy was shaped around a replicable and spreadable intervention. The change in technological component, however, created new unanticipated interdependencies between the *wider context* (NASSS domain 6), *organisation* (NASSS domain 5), and *technology* (NASSS domain 2) exposing a lack of infrastructure. Infrastructure broadly encompasses material features (hardware, software, servers, interfaces, and connectivity) and governance processes (external and internal laws, rules, and standards) that have to be managed over time by multiple stakeholders [29–31]. Piloting digital interventions in ‘best-case’ settings, like SMASH, meant these interdependencies were unanticipated. This suggests that future piloting of digital interventions should include ‘worst-case’ settings to understand infrastructure needs and the work and time needed to scale up the intervention.

The longitudinal study approach is a strength of the study, offering a perspective of the meso- and macro-factors influencing implementation enactment that is often overlooked in the literature [32]. Data collection over three years allowed an exploration of how expectations and management of SMASH developed over time, and the adaptations needed to try and make it work, leading to some recommendations. First, the findings provide support for distinguishing the role of pilots for efficacy and pilots for implementation [10]. Second, there should be greater consideration of the implications of changing or modernising the technological component of an existing intervention in healthcare. A limitation of the findings, however, is that due to the study timelines, the focus is almost exclusively on the implementation of the technological component of SMASH. Work to understand how the workforce adopts, tinkers with, or resists both the social and technological components of SMASH is needed.

## Conclusion

The role of innovation organisations in translating interventions into practice is increasingly the focus of research examining implementation in healthcare settings. This paper highlights the importance of agreed roles and responsibilities between the organisation and the localities it works with in coordinating digital intervention implementations. Increasingly complex digital interventions, such as those involving health data sharing, may need greater time and resources to scale up infrastructure at the outset for better synchronisation of implementation. Social science approaches to time provide a theoretical starting point for future research on understanding the role of pace for implementation.

## Supplementary Information

Below is the link to the electronic supplementary material.

Supplementary Material 1

## Data Availability

The data supporting the findings of this study are not publicly available to safeguard the privacy and confidentiality of the participants.
