# Supplementary material for: A longitudinal qualitative evaluation of the rollout of a complex digital intervention across healthcare organisations and the significance of implementation pace
Source: BMC Health Serv Res. 2026 Jul 21;26:1422. doi: 10.1186/s12913-026-15183-w (PMC13679135; doi:10.1186/s12913-026-15183-w)
Supplement: Supplementary file 1 [file 12913_2026_15183_MOESM1_ESM.docx]

**SMASH Evaluation - Topic Guide**

**Interview questions:**

1. Can you tell me a little about your current role and what this involves?
2. What role and involvement have you had with the Safe Medication Dashboard (SMASH)?
3. Did you have any prior awareness/knowledge/experience of SMASH before its implementation?

*Prompts:* What information? From local or regional organisations? When?

*Follow up.* As such, what were your expectations about SMASH and its implementation from this?

4. Have you experienced any barriers to implementing SMASH in your organisation?

*Follow up*: Were these expected or unexpected? How were these managed?

5. Have you experienced any support/ resources that have helped to facilitate implementing SMASH in your organisation?

*Follow up*: Were these expected or unexpected? How were these managed?

6. Can you tell me about the role of any other organisations that you came across that were involved in the implementation of SMASH

*Prompts:* regional organisation, external providers, others)?

7. What do you believe are the next steps needed to implement SMASH across the organisation?
8. How confident are you that SMASH can be sustained in your organisation going forward?

*Prompts*: What makes you say that?

9. What do you consider to be the main risks to the sustainability and development of SMASH going forward?
10. How confident are you that SMASH will contribute to reducing hazardous prescribing within your organisation?
11. Is there anything else you think is important that we haven’t covered relating to the implementation of SMASH?
